# Integration of RNA Editing into Multiomics Machine Learning Models for Predicting Drug Responses in Breast Cancer Patients

**DOI:** 10.3390/biomedicines14030665

**Published:** 2026-03-14

**Authors:** Yanara A. Bernal, Alejandro Blanco, Karen Oróstica, Iris Delgado, Ricardo Armisén

**Affiliations:** 1 Centro de Genética y Genómica, Instituto de Ciencias e Innovación en Medicina, Facultad de Medicina Clínica Alemana Universidad del Desarrollo, Santiago 7550000, Chile; yanara.bernal@uc.cl (Y.A.B.); ablanco@udd.cl (A.B.); 2 Nursing School, Faculty of Medicine, Pontificia Universidad Católica de Chile, Santiago 7820436, Chile; 3 Institute of Data Science, Universidad del Desarrollo, Santiago 7550000, Chile; korostica@udd.cl; 4 Centro de Epidemiología y Políticas Públicas, Instituto de Ciencias e Innovación en Medicina, Facultad de Medicina Clínica Alemana Universidad del Desarrollo, Santiago 7550000, Chile; idelgado@udd.cl

**Keywords:** precision medicine, machine learning, multi-omics, RNA editing, breast cancer, drug response

## Abstract

**Background**: The integration of multi-omics data, such as genomics and transcriptomics, into artificial intelligence models has advanced precision medicine. However, their clinical applicability remains limited due to model complexity. We integrated DNA mutation, RNA expression, and A>I(G) RNA editing data to develop a predictive model for drug response in breast cancer. **Methods**: We analyzed 104 patients from the Breast Cancer Genome-Guided Therapy Study (ClinicalTrials.gov: NCT02022202). Clinical variables, gene expression, tumor and germline DNA variants, and RNA editing features were integrated into machine learning models to predict therapy response. Generalized linear models (GLM), random forest (RF), and support vector machines (SVM) were trained and evaluated across multiple random 70/30 train-test splits. Feature selection was performed exclusively within the training set using LASSO regularization. Model performance was assessed using the F1-score on independent test sets. The additive effect of RNA editing was evaluated using paired comparisons across identical train/test splits. **Results**: We characterized the cohort using clinical, mutational, transcriptomic, and RNA editing profiles in 69 non-responders and 35 responders. Across repeated splits, adding RNA editing frequently maintained or modestly improved predictive performance, particularly in expression-based models, with paired analyses showing a statistically significant increase in F1-score. **Conclusions**: RNA editing represents a complementary molecular layer that can enhance multi-omic models for therapy response prediction in breast cancer, supporting further investigation of epitranscriptomic features in precision oncology.

## 1. Introduction

The integration of artificial intelligence (AI) into clinical decision-making holds immense promise for enhancing health outcomes across diverse diseases, including cancer [1]. AI tools have demonstrated potential in early diagnosis, comprehending intricate biological mechanisms, and facilitating the development of novel therapeutic strategies [2]. However, the successful translation of AI models into clinical practice for cancer treatment faces substantial challenges. The heterogeneity of the response to anticancer drugs and the development of therapeutic resistance represent significant clinical challenges, leading to increased mortality rates worldwide [3,4]. Resistance to therapy in breast cancer (BC) is multifactorial, with contributing mechanisms including increased drug efflux, alterations in the tumor microenvironment, epithelial—mesenchymal transition, tumor heterogeneity, therapeutic target alterations, adaptive responses, and DNA damage repair [4,5,6,7]. Between 30% and 50% of BC patients may develop therapy resistance, resulting in a drastically reduced survival time of 2 to 3 years compared with 5 years in responders [8]. Therefore, early prediction of the therapeutic response is crucial for timely and effective clinical decision-making.

Recent advancements have integrated conventional omics data, such as germline and tumoral DNA mutation data and RNA expression data, into AI drug response models. Nevertheless, these approaches often overlook crucial factors influencing tumor complexity. This limitation, compounded by methodological issues such as poor data quality, missing data, and small sample sizes, contributes to the difficulty in replicating cancer study findings across independent cohorts [9]. These factors introduce biases into AI predictive models, complicate the interpretation of machine learning’s “black box” concept, and hinder the translation of AI models into clinical practice [10,11].

In this context, RNA editing, a posttranscriptional modification mediated by ADAR enzymes, presents a promising avenue to address some of these challenges. This process, involving the conversion of adenosine to inosine (A>I(G)) in RNA, can significantly impact gene product structure and function, influencing tumor biology and drug response [12]. Despite its potential relevance, research on RNA editing in cancer, particularly its integration into AI models, remains limited. While pancancer studies have described certain RNA-edited sites [13] and preliminary work has explored the role of RNA editing in the drug response of patients with BC via cell lines [14,15], its clinical implications remain largely unexplored. Notably, RNA editing has not been systematically incorporated into AI models for predicting clinical outcomes in cancer [16].

Recent studies have highlighted the potential of RNA editing-based predictive models in various cancers, including gastric cancer [17], lung cancer [18], acute myeloid leukemia (AML) [19], and lower-grade gliomas [20]. However, the utilization of RNA editing for predicting drug response in BC remains underexplored.

This study addresses these gaps by integrating multi-omics data with a specific focus on A>I(G) RNA editing to evaluate its additive contribution to the prediction of therapy response in BC. Using clinical trial data, we systematically compare machine learning models trained with and without RNA editing features across identical train/test splits to quantify their impact on predictive performance. By combining RNA editing with gene expression, DNA variants, and clinical variables, our results highlight RNA editing as a biologically relevant and underexplored molecular layer that enhances multi-omics machine learning models and supports more accurate and reproducible prediction of therapy response in BC.

## 2. Materials and Methods

### 2.1. Dataset and Breast Cancer Patients

One hundred and four patients were analyzed from the Breast Cancer Genome-Guided Therapy Study (ClinicalTrials.gov: NCT02022202) out of one hundred and eighteen BC patients according to data availability. The clinical characterization of these patients was based on therapy response, which was defined as a response to therapy when reported as a pathological complete response in the breast and nodes (path-CR) after 24 weeks of chemotherapy (adriamycin and cyclophosphamide or epirubicin and cyclophosphamide or 5-flurouracil, epirubicin and cyclophosphamide), whereas nontherapy response referred to when there was no pathological complete response [21,22]. Additionally, the molecular subtype was defined based on baseline Ki67 results, estrogen receptor levels, and HER2 status (by immunohistochemistry (IHC) or fluorescence in hybridization (FISH)) from the original study.

### 2.2. Whole Exome Sequencing (WES) Analysis and Variant Calling

WES data from paired tumor and normal samples were analyzed via an automated pipeline deployed on the SevenBridges cloud platform (https://www.sevenbridges.com/). The raw sequencing reads in FASTQ format underwent initial processing with Trim Galore v0.6.10 [23] to remove low-quality bases and adapter sequences, ensuring high-quality input for downstream analysis. The trimmed FASTQ files were then converted into unmapped BAM (uBAM) format via Picard’s FatsqToSam tool v3.0.0 [24], which added the read group information necessary for alignment. The uBAM files were subsequently aligned to the GRCh38 reference genome via BWA-MEM [25]. Following alignment, the BAM files were processed following GATK v4.2.0 Best Practices [26,27,28] to produce high-quality analysis-ready BAM files. This included marking duplicate reads with Picard’s MarkDuplicates to mitigate biases from PCR amplification and performing base quality score recalibration (BQSR) via GATK’s BaseRecalibrator and ApplyBQSR, incorporating known variant sites to ensure accuracy.

Somatic variants were identified via GATK Mutects2 v4.2.5 in tumor-normal mode. The matched normal samples were utilized to distinguish somatic mutations from germline variants and sequencing artifacts. GATK’s FilterMutectCalls was applied to refine the somatic variant calls further. Germline variants were called via GATK’s v4.2.0 HaplotypeCaller in GCVF mode on the normal samples. The resulting gVCFs were combined via CombineGVCFs, and the joint genotyping step was performed with GenotypeGVCFs to produce a multisample VCF. The genotyped VCF was filtered using GATK’s VariantRecalibrator and ApplyVQSR separately for both SNPs and InDels. The VCFs were subsequently split into individual VCFs to facilitate downstream analyses. Annotation of both somatic and germline variants was conducted via the Ensembl Variant Effect Predictor (VEP) v112 [29], which adds functional and clinical information, including gene impact, variant consequences, and pathogenicity predictions. Finally, the annotated VCFs were converted into mutation annotation format (MAF) files via the vcf2maf tool v1.6.21 [30] to enable compatibility with downstream analysis.

### 2.3. RNA-Seq Analysis

The RNA sequencing data were preprocessed and analyzed via the nf-core/rnaseq pipeline (v3.14.0) implemented in NextFlow (v23.04.2). The analysis was performed with GRCh38 as the reference genome and followed standard best practices for RNA-seq data analysis. Initially, raw FASTQ files were subjected to quality control and adapter trimming via Trim Galore v0.6.7, ensuring that low-quality bases and adapter sequences were removed. Trimmed FASTQ files were then aligned to the reference via STAR v2.7.9a in two-pass mode, which improves splicing accuracy by using junction information obtained from the first pass during the second pass of alignment. Salmon v1.10.1 quantification was performed alongside STAR alignment to estimate transcript abundance via quasimapping and expression quantification. Gene annotation for alignment and quantification was based on the GENCODE v43 annotation file, ensuring compatibility with the reference genome. Multiple quality control steps were performed on the BAM files via RSeQC v5.0.2, SAMtools v1.17, Dupradar (r bioconductor v1.28.0) and Qualimap v2.3 to ensure the integrity of the data. MultiQC v1.19 [31] was used to report the results.

### 2.4. Tumoral and Germline DNA Variant Characterization

To focus the analysis on biologically and clinically validated cancer-relevant alterations, reduce dimensionality, and mitigate the risk of overfitting in our machine learning models, we restricted the consideration of tumor variants exclusively to those reported in genes listed in the Cancer Gene Census (CGC) from COSMIC V100 https://cancer.sanger.ac.uk/cosmic/download/cosmic (accessed on 10 March 2026) [32]. We evaluated differences per variant and gene mutation in the responder and nonresponder groups via Fisher’s exact test for germline mutations and focused on genes related to high-risk cancer predisposition: ATM, BAPI, BMPR1A, BRCA1, BRCA2, BRIP1, MSH2, MSH6, MUTYH, DICER1, PALB2, RUNX1, SDHAF2, SDHB, SDHC, and SDHD as Tier 1 of high risk; Tier 2: APC, CDH1, MLH1, MEN1, NF1, NF2, PMS2, POLE, PTEN, PTPN11, RB1, RET, SMAD4, SMARCA4, STK11, TGFBR2, TSC1, TSC2, VHL and WT1 as intermediate risk; and Tier 3 BARD1, CHECK2, HNF1A, FH, NBN, RAD50, RECQL4, and TP53 [33].

### 2.5. Gene Expression Abundance Estimation

Differential expression analysis (DEA) between the response and non-response groups was performed via the raw transcript-level quantification files generated by Salmon during the nf-core/rnaseq analysis [34]. For visualization and exploratory purposes, to enable the use of DESeq2 (r bioconductor v1.28.0) [35] for differential expression analysis, the transcript quantification values were approximated to the nearest integer. Differentially expressed transcripts were visualized via a volcano plot generated with EnhancedVolcano, applying a *p*-adjusted cutoff of <0.05 and a fold-change (FC) threshold of >2.5. For the creation of predictive models, Salmon’s gene-level quantification files normalized to transcripts per million (TPM) were utilized. For predictive modeling, gene expression features were represented by TPM-normalized gene-level values and included directly in the machine learning pipeline, where all feature filtering and selection steps were performed exclusively within the training set to prevent information leakage. All analyses and plots were constructed using R version 4.2.2 (31 October 2022), dplyr package version 1.1.3, FactoMineR package version 2.11, Deseq2 package version 1.38., and Caret package version 6.0.94.

### 2.6. High-Confidence RNA Editing Identification

REDITools2 was used to identify RNA-edited sites on the basis of a previously published methodology, which briefly consisted of BAM files from STAR alignment in nf-core/rna-seq [36,37]. After applying REDITools to all the BAM files, we excluded all sites found as mutations A/G or T/C from the DNA variants called in the tumor and/or germline. For RNA-edited site identification, we consider only sites that are the reference/alternate of A/G or T/C. For these sites, we calculated the RNA editing level at each site, which consists of the ratio between mismatch (A/G on the positive strand or T/C on the negative strand) reads and total readings at the site (both mismatch and match, represented by A/A on the positive strand or T/T on the negative strand). The RNA-edited level per site was included in the models. Additionally, differential RNA editing analyses between responders and non-responders were performed as an exploratory characterization using RNA-editing tests (REDITs) to identify RNA-edited sites between responders and non-responders via the beta-binomial distribution for characterization and selection of RNA-edited sites (FC cutoff > 0.05 and *p* adjust < 0.01) [38]. For machine learning analyses, RNA editing levels were included as candidate features, and all feature selection steps were carried out exclusively within the training set.

### 2.7. Predictive Models

Predictive models were developed to assess the contribution of RNA editing features to therapy response classification. The study cohort consisted of 104 patients from the Breast Cancer Genome-Guided Therapy Study with complete multi-omics and clinical data. Clinical variables included molecular subtype, histological type, TNM stage, and age group, and were included in all models. Molecular features were derived from three data types: gene expression (EXP), RNA editing sites (ED), and tDNA/gDNA (DNA). RNA editing features were represented as continuous editing levels per site, while gene expression features corresponded to normalized transcript abundance values.

To prevent information leakage, all feature selection steps were performed exclusively within the training set for each model iteration. Final feature selection and model fitting were then performed using least absolute shrinkage and selection operator (LASSO) regularization. The dataset was split into training (70%) and test (30%) subsets, and this procedure was repeated across multiple random seeds to evaluate model stability. Within each training set, 10-fold cross-validation was used for hyperparameter tuning and model optimization. Three machine learning algorithms were evaluated: generalized linear models (GLM), random forest (RF), and support vector machines (SVM).

Model performance was assessed on the independent test set using accuracy, precision, recall, and F1-score, with F1-score selected as the primary metric due to class imbalance. Performance variability was evaluated across repeated train/test splits, and confidence intervals for the F1-score were derived from the empirical distribution obtained across random seeds. In addition to F1-score, model performance was further characterized using the Matthews correlation coefficient (MCC) and precision–recall area under the curve (PR-AUC) as complementary metrics robust to class imbalance. All analyses were conducted using therapy response as the primary outcome, and an additional complementary analysis was performed defining non-response as the positive class to evaluate the model’s ability to identify patients at risk of treatment failure. To evaluate the additive effect of RNA editing, models trained with RNA editing features were compared to their corresponding models without RNA editing using paired analyses based on identical train/test splits. The following feature combinations were evaluated:

Model 1: Therapy response ~ Clinical + Gene Expression (EXP)
Model 2: Therapy response ~ Clinical + Gene Expression (EXP) + **RNA editing (ED)**
Model 3: Therapy response ~ Clinical + tDNA/gDNA (DNA)
Model 4: Therapy response ~ Clinical + tDNA/gDNA (DNA)+ **RNA editing (ED)**
Model 5: Therapy response ~ Clinical + Gene Expression (EXP) + tDNA/gDNA (DNA)
Model 6: Therapy response ~ Clinical + Gene Expression (EXP) + tDNA/gDNA (DNA) + **RNA editing (ED)**

**Figure 1:**
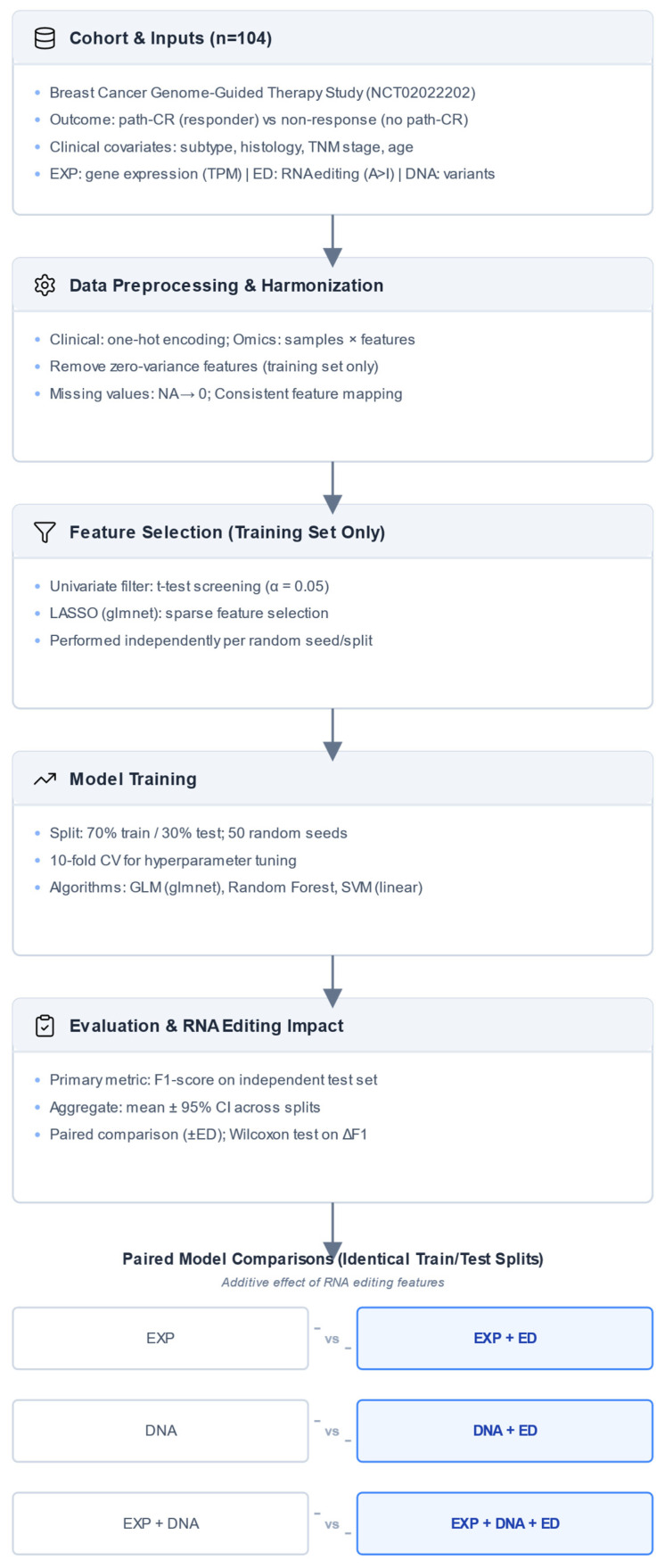
Workflow for integrating RNA editing into multi-omics machine learning models for drug response prediction in breast cancer. The schematic illustrates the full analytical pipeline applied to the cohort of 104 breast cancer patients. Clinical variables, gene expression (EXP), tumor and germline DNA variants (DNA), and RNA editing levels (ED) were preprocessed and aligned at the sample level. All feature filtering and selection steps, including univariate screening and LASSO regularization, were performed exclusively within the training set for each split to prevent information leakage. Models were trained using generalized linear models (GLM), random forest (RF), and support vector machines (SVM) with repeated 70/30 train-test splits and 10-fold cross-validation within the training set. Model performance was evaluated on independent test sets using the F1-score. The additive contribution of RNA editing was assessed through paired comparisons between models trained with and without RNA editing features across identical train/test splits, enabling direct evaluation of its impact on predictive performance.

Although several classifiers achieved comparable performance in terms of F1-score, we chose the final model based on its stability across random seeds, consistent performance across multiple training and testing splits, and the potential for direct interpretability of feature effects. This interpretability would allow us to identify clinically, transcriptionally, and RNA editing-related features that are relevant to predicting therapeutic response (Figure 1).

## 3. Results

### 3.1. Clinical and Molecular Characterization of Cohort

The clinical characteristics analyzed included molecular subtype, tumor size, nodal status, histological type, and age group. Molecular subtype was significantly different between responders and non-responders to therapy (*p* < 0.001), whereas other variables, such as tumor size, nodal status, histological type, and age, were not significantly different (Figure S1).

**Figure 2:**
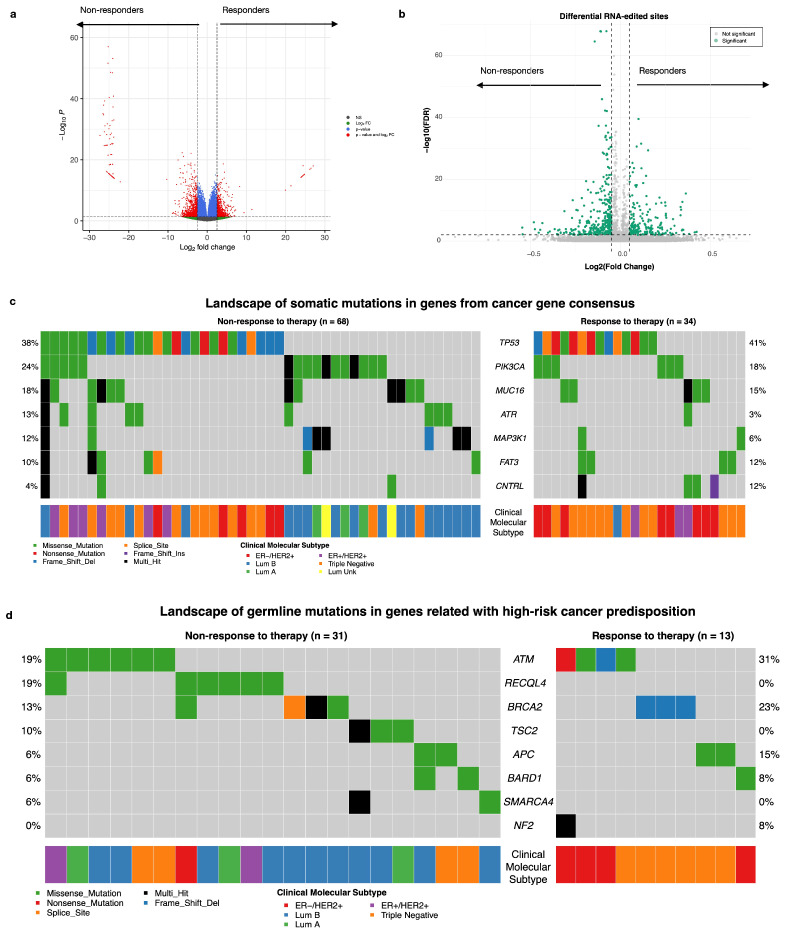
Exploratory clinical and molecular landscape of cohort. (**a**) Differential expression transcript in Volcanoplot FC cutoff > 2.5 and *p*-adjust cutoff < 0.05; (**b**) Differential RNA-edited level in Volcanoplot FC cutoff > 0.05 and *p*-adjust cutoff < 0.01; (**c**) landscape of somatic mutations in genes from the Cancer Gene Census (CGC) and (**d**) landscape of germline mutations in genes related to high-risk cancer predisposition in Oncoplot by drug response, each row represents a gene, and each column represents a patient with at least one variant (*n* = 44 subjects). The colors indicate different types of mutations and the molecular subtypes of the patients.

As an exploratory analysis aimed at biological characterization of the cohort, we performed differential transcript expression analysis, and we identified 996 differentially expressed genes. We highlighted transcripts of SNX14, RHOT2, PIK3R1, SLC7A4, DTNA, and even RAD51 in the non-responders, whereas genes such as IFITM3, CYP2T1P, TMUB2, and PAX6 were prominent in the responders (Figure 2a). However, we did not find significant differences in ADAR1 (ENST00000492630.2) expression between the groups (Table S1). Similarly, in an exploratory analysis of RNA editing, 500 sites were identified as significantly different between responders and non-responders to therapy. Among these, we highlight specific sites within genes such as ALPL (COSV66379629), DHTKD1, ABCC4 (COSV65312135), GAA (COSV56406822), USP34, ZNF662, and NFKBIZ (COSV58198879). These sites result in missense mutations, have been previously reported in the COSMIC database, and are predicted to be potentially damaging by PolyPhen and deleterious by SIFT (Figure 2b and Table S2). Among the 290 somatic variants identified in the cancer gene consensus (CGC) cohort, the most altered genes in non-responders were somatic variants in TP53, PIK3CA, and MUC16, whereas responders presented mutations in genes such as ATR, MAP2K1, and FAT3 (Figure 2c). We did not find significant differences by gene or by variant between the responder and non-responder groups. (Tables S3 and S4). In terms of germline mutations, we selected 47 variants from the list of high-risk cancer predisposition genes; only 44 patients (42.31%) had at least one germline mutation. Notably, responder patients presented alterations in genes such as ATM, RECQL4, and BRCA2, whereas APC and NF2 were prominent in non-responders (Figure 2d). These differential analyses were conducted solely for cohort characterization and biological interpretation and were not used for feature selection in the predictive modeling analyses described below.

### 3.2. Machine Learning Models for Drug Response

**Figure 3:**
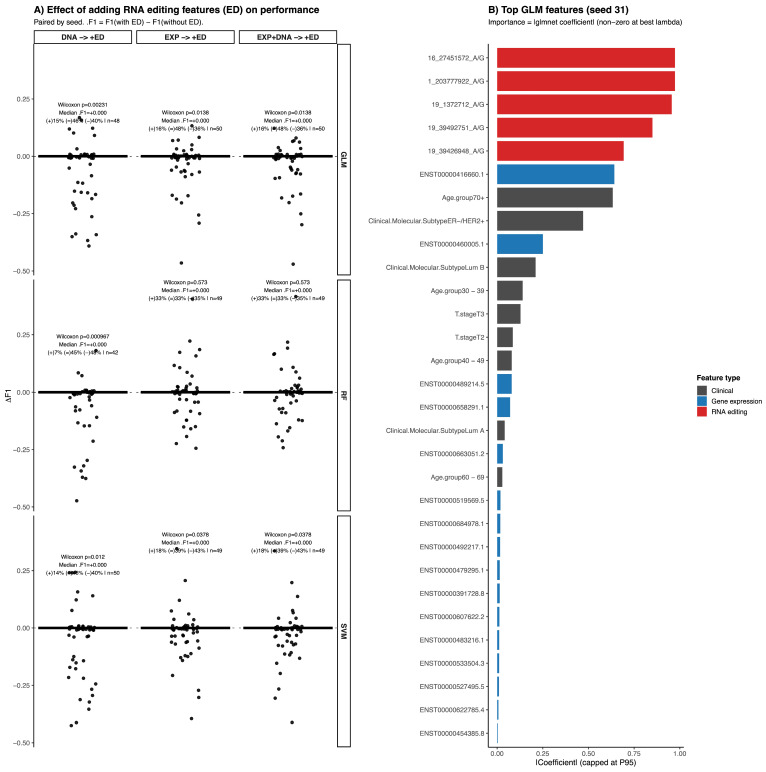
Effect of adding RNA editing features on model performance in drug response analysis. (**A**) Paired change in F1-score (ΔF1 = F1 with ED − F1 without ED) across repeated train/test splits (seeds) when adding RNA editing features to DNA-only, EXP-only, and EXP + DNA feature sets for GLM, RF, and SVM models. Points represent individual seeds (paired splits). Paired Wilcoxon *p*-values, median ΔF1, and the proportion of seeds with improved (+), unchanged (=), or worsened (−) performance are shown in each panel. (**B**) Top GLM features for the representative split (seed 31), ranked by absolute glmnet coefficient (|β|, capped at the 95th percentile). Features are classified as clinical, gene expression, or RNA editing.

To determine the added predictive value of RNA editing features in therapy response classification, we evaluated multiple machine learning algorithms using repeated random train/test splits and systematically compared models trained with and without RNA editing information. Paired comparisons across identical splits revealed modest and variable changes in F1-score when RNA editing features were included (paired Wilcoxon test; Figure 3A). Although some splits showed improvement, the overall median ΔF1 was small, indicating that the performance gain was not consistent across all data partitions. Considering both performance distribution and model stability, the regularized GLM integrating gene expression and RNA editing features (ED_EXP) was selected for further analysis. Across 50 independent train/test splits, this model achieved a mean F1-score of 0.49 (median = 0.5), with a 95% confidence interval of 0.26–0.69 and a standard deviation of 0.11. To further evaluate model behavior, we performed an additional analysis in which the positive class was defined as non-response to therapy, reversing the outcome encoding used in the main analysis. Under this formulation, the model showed a mean F1-score of 0.754 ± 0.05, with a 95% confidence interval of 0.67–0.825, a ROC-AUC of 0.69 ± 0.08 and a PR-AUC of 0.83 ± 0.06, the model showed adequate sensitivity for identifying non-responders (0.79 ± 0.1), while specificity was lower (0.41 ± 0.15), resulting in a balanced accuracy of approximately 0.6, indicating moderate performance across both response classes (Figures S3 and S4, Table S6). Consistent with the main analysis, paired comparisons across identical splits showed small median changes in F1-score after adding RNA editing features (median ΔF1 ≈ 0). While some comparisons reached statistical significance, the magnitude of the effect remained small, and a substantial proportion of splits showed either improved or unchanged performance, indicating variable but generally modest contributions of RNA editing features to model performance (Figure S3).

Feature importance analysis in the selected GLM model showed that RNA editing events ranked among the most informative predictors alongside gene expression and clinical variables (Figure 3B), highlighting their complementary contribution to therapy response prediction. This split is shown to illustrate model behavior and feature contribution, rather than to represent average performance. MCC and PR-AUC analyses showed patterns consistent with the F1-score distribution. While the MCC displayed moderate variability across splits, PR-AUC remained relatively stable, collectively supporting the robustness of the selected ED_EXP GLM model (Figure S5).

Feature importance analysis in the GLM corresponding to the selected representative split revealed that RNA editing events dominated among the highest-contributing predictors (Figure 3B; Table S5). The top five ranked features were all RNA editing sites: Chr1:203777922 A/G (LAX1, downstream), Chr16:27451572 A/G (IL21R, 3′UTR), Chr19:1372712 A/G (PWWP3A, intron), Chr19: 39492751 A/G (TIMM50, 3′UTR), and Chr19:39426948 A/G (PLEKHG2, 3′UTR). Among these, the editing event in PWWP3A (rank 3) showed a positive coefficient and an odds ratio of 2.6, indicating an increased risk of non-response. In contrast, the remaining four top-ranked RNA editing events displayed negative coefficients and odds ratios below 1, suggesting a reduced risk of non-response.

Gene expression features appeared from rank 6 onward and generally exhibited smaller effect sizes, with odds ratios close to 1. Clinical variables contributed more modestly, although Luminal B subtype, age group 30 to 39 years, and stage III showed positive associations with non-response. Overall, the predominance of RNA editing events among the highest-ranked predictors in this representative split supports their substantial and complementary contribution to therapy response prediction.

## 4. Discussion

The present study aimed to evaluate whether RNA editing provides additional predictive value in multi-omic models of therapeutic response in breast cancer. By systematically comparing models trained with and without RNA editing features across repeated train/test splits, we observed that the inclusion of RNA editing rarely degraded model performance and occasionally improved it. Although the magnitude of performance gain varied across data partitions, RNA editing did not diminish model stability, supporting its role as a complementary molecular layer in therapy response prediction.

From a performance perspective, the use of the F1-score as the primary metric allowed for a balanced evaluation of precision and recall under class imbalance. In the main formulation predicting therapy response, the model achieved a mean F1-score of approximately 0.49 across repeated train/test splits. When the outcome encoding was reversed and non-response was defined as the positive class, overall discrimination remained comparable, with improved sensitivity for identifying non-responders and stable PR-AUC and MCC distributions. This consistency across outcome definitions indicates that the model captured similar predictive patterns when response or non-response was used as the positive class. Notably, defining non-response as the positive outcome improved sensitivity for identifying patients who may fail therapy.

Across the evaluated algorithms, GLM exhibited the most stable and interpretable improvement following the inclusion of RNA editing features, particularly in models incorporating gene expression data. These results support the notion that RNA editing captures complementary biological signals not fully represented by steady-state gene expression or DNA variation alone. In contrast, models trained solely on clinical or DNA mutation data showed lower performance, reinforcing previous observations that single-omic approaches are insufficient to capture the complexity of therapeutic response in BC [39].

Across evaluated algorithms, the regularized GLM showed the most consistent and interpretable behavior after integrating RNA editing features, particularly in the combined ED_EXP dataset. In the representative split selected for detailed analysis, RNA editing events dominated the highest-ranked predictors, with the top five features corresponding exclusively to editing sites in LAX1 (downstream), IL21R (3′UTR), PWWP3A (intron), TIMM50 (3′UTR), and PLEKHG2 (3′UTR). This predominance suggests that epitranscriptomic variation captures regulatory signals not fully reflected by steady-state gene expression alone. Although the direct role of some of these genes in breast cancer remains to be fully elucidated, they are linked to pathways such as immune signaling, mitochondrial regulation, and cytoskeletal remodeling, which are broadly associated with tumor progression and therapeutic resistance. IL21R encodes the receptor for interleukin-21, a cytokine involved in immune regulation within the tumor microenvironment. Although its direct role in breast cancer progression remains incompletely defined, IL-21 signaling has been implicated in modulating antitumor immune responses, suggesting that post-transcriptional variation in IL21R may influence tumor–immune dynamics [40]; TIMM50 has been reported to be overexpressed in BC and to regulate cell proliferation and apoptosis through modulation of mitochondrial membrane potential [41]; PWWP3A has been identified as a regulator of innate immune signaling, where it modulates the assembly of the VISA/MAVS signalosome and controls type I interferon responses [42], given the emerging crosstalk between DNA damage response, innate immunity, and tumor progression, regulatory variation in PWWP3A may influence immune-related mechanisms that are increasingly recognized as critical determinants of therapeutic response; PLEKHG2 encodes a Rho guanine nucleotide exchange factor involved in Rac and Cdc42 signaling, and has been associated with EGFR-mediated pathways in triple-negative breast cancer. High PLEKHG2 expression has been linked to worse recurrence-free survival in basal-like TNBC, supporting its role in tumor progression and signaling dysregulation [43]. Together, these findings indicate that RNA editing features enhance predictive models not merely by increasing dimensionality but by capturing regulatory variability in pathways related to immune modulation, mitochondrial function, chromatin organization, and cellular plasticity mechanisms closely associated with therapeutic resistance and sensitivity.

Importantly, while RNA editing frequently maintained or improved performance across feature combinations, its impact was not uniform across all datasets. This heterogeneity suggests partial overlap with gene expression–derived signals and underscores the importance of parsimonious feature selection strategies such as LASSO to mitigate redundancy in high-dimensional multi-omic models.

Several limitations should be considered when interpreting these findings. A limitation of this study is the relatively modest sample size (*n* = 104). In high-dimensional multi-omics machine learning, where the number of features vastly exceeds the number of samples (*p* >> *n*), traditional *a priori* statistical power calculations are challenging to apply directly [44]. To address the inherent risk of model instability and overfitting in this setting, we evaluated the adequacy of our sample size empirically. We employed rigorous resampling techniques, specifically 50 repeated random train/test splits combined with internal 10-fold cross-validation [45], alongside LASSO regularization [46]. This iterative approach allowed us to generate empirical confidence intervals for our performance metrics, demonstrating that the cohort possessed sufficient information to detect a reproducible predictive signal.

Although repeated train/test splits and paired comparisons were used to reduce overfitting and assess robustness, the observed improvements in F1-score should be regarded as indicative rather than definitive. In addition, no independent external validation cohort was available, and RNA editing detection is highly sensitive to technical factors related to library preparation, sequencing depth, alignment strategies, and variant calling algorithms, which complicates reproducibility across studies [47,48]. Furthermore, while our RNA-seq data derived from a single clinical trial processed through standardized nf-core pipelines mitigates massive technical divergence, we did not explicitly model or correct for unmeasured batch effects. Addressing potential batch effects and standardizing normalization techniques will be a priority for future studies. Given the relatively modest sample size and the absence of an independent external validation cohort, our findings should be interpreted as a proof-of-concept demonstrating the feasibility of incorporating RNA editing into multi-omic predictive models, rather than as a clinically deployable predictive tool. Future validation strategies must include independent prospective cohorts processed with harmonized pipelines, alongside targeted low-throughput approaches such as RNA editing site-specific quantitative PCR (RESqPCR) to biologically confirm the candidate editing events identified by our models. Therapeutic response was modeled as a binary outcome encompassing heterogeneous treatment regimens, and the lack of detailed drug-specific information restricts the ability to predict responses to individual therapies and may obscure treatment-specific signals. While the inclusion of RNA editing features consistently improved predictive performance, the magnitude of this improvement was moderate, suggesting that RNA editing provides complementary rather than dominant predictive information and that part of its signal may overlap with gene expression and clinical features, particularly in more complex feature combinations [49]. Finally, although feature importance analyses highlighted biologically plausible genes involved in therapeutic resistance, DNA repair, and tumor plasticity, the observational nature of the study precludes causal inference, and the identified RNA editing events and transcripts should therefore be interpreted as predictive biomarkers rather than direct drivers of treatment response [50].

## 5. Conclusions

In conclusion, our results indicate that integrating RNA editing with gene expression and clinical data can contribute complementary information to machine learning models for drug response prediction in breast cancer. Across repeated train/test splits, the inclusion of RNA editing features generally maintained model performance and, in some configurations, provided modest improvements in the discrimination of non-responders. Within the regularized GLM framework, RNA editing features appeared among the highest-ranked predictors in the representative split, suggesting potential regulatory relevance within multi-omic predictive models. Rather than merely increasing dimensionality, RNA editing appears to capture complementary regulatory variability partially overlapping with gene expression-derived signals, particularly in pathways related to immune modulation, mitochondrial function, and cellular plasticity. Overall, these findings support the exploration of RNA editing as an additional molecular layer in integrative modeling strategies and provide a rationale for further investigation of epitranscriptomic variation in precision oncology.

## Data Availability

The data used in this study is available from the publicly dbGaP repository under the study accession number phs001050.v1.p1. The codes are deposited at https://github.com/ybernalg/RNA_editing_multiomic_machinelearning_models_breastcancer (accessed on 10 March 2026). Additional details about the codes are available from the corresponding author upon reasonable request.

## Supplementary Materials

The following supporting information can be downloaded at: https://www.mdpi.com/article/10.3390/biomedicines14030665/s1, Table S1: Results of differential expression analysis between responders and non-responders to therapy; Table S2: Results of differential RNA editing site analysis between responders and non-responders to therapy; Table S3: Results of tumor mutation differences per gene between responders and non-responders to therapy; Table S4: Results of tumor variant differences between responders and non-responders to therapy; Table S5. Top features of the final GLM model for predicting therapy non-response. The most informative predictors selected in the final GLM, integrating RNA editing (ED), gene expression (EXP), and clinical variables. For each feature, the corresponding genomic or transcript annotation, omics category, model coefficient (β), odds ratio (OR = e^β), and direction of effect on non-response risk are reported. Positive coefficients (OR > 1) indicate an increased risk of non-response to therapy, whereas negative coefficients (OR < 1) indicate a reduced risk. Features are ranked according to the absolute value of their coefficients, reflecting their relative importance in the model; Table S6. Performance metrics of the ED_EXP GLM model when non-response to therapy was defined as the positive class. Metrics were calculated across 50 independent train/test splits. Values represent mean ± SD. Abbreviations: ROC-AUC, area under the receiver operating characteristic curve; PR-AUC, area under the precision–recall curve; MCC, Matthews correlation coefficient; Figure S1. Clinical and molecular characterization of the training and testing cohorts. (a) Table of clinical characterization by drug response. *p*-values were obtained using the chi-square test (or Fisher’s exact test when expected cell counts were <5); Figure S2. Model performance across datasets. Mean F1-score ± 95% confidence interval across datasets for generalized linear models (GLM), random forest (RF), and support vector machines (SVM). Feature combinations include: gene expression (EXP), tumor and germline DNA variants (DNA), RNA editing (ED), gene expression plus DNA (EXP_DNA), gene expression plus RNA editing (ED_EXP), DNA plus RNA editing (ED_DNA), and the full multi-omic integration of gene expression, DNA, and RNA editing (ED_EXP_DNA); Figure S3. Effect of RNA editing features on model performance in the non-response analysis. (A) Change in F1-score (ΔF1 = F1 with ED − F1 without ED) across 50 paired train/test splits when adding RNA editing features to DNA-only, EXP-only, and EXP + DNA models using GLM, RF, and SVM algorithms. Points represent individual splits. (B) Top GLM features ranked by absolute glmnet coefficient (|β|) for a representative split. Features are classified as clinical, gene expression, or RNA editing; Figure S4. Model performance when predicting non-response to therapy. Performance of GLMNET, RF, and SVM models across 50 train/test splits when non-response was defined as the positive class. Metrics shown include balanced accuracy, F1-score, PR-AUC, and ROC-AUC; Figure S5. Distribution of MCC and PR-AUC across repeated train/test splits for the selected ED_EXP GLM model. Boxplots represent the distribution of Matthews correlation coefficient (MCC) and precision–recall area under the curve (PR-AUC) across 50 independent random 70/30 train/test splits. Points correspond to individual seeds. The black dot indicates the mean, and error bars represent mean ± standard deviation.
