# Supplementary material for: Integration of RNA Editing into Multiomics Machine Learning Models for Predicting Drug Responses in Breast Cancer Patients
Source: Biomedicines. 2026 Mar 14;14(3):665. doi: 10.3390/biomedicines14030665 (PMC13024426; doi:10.3390/biomedicines14030665)
Supplement: Supplementary file 1 [file biomedicines-14-00665-s001.zip › Figure S3.pdf]

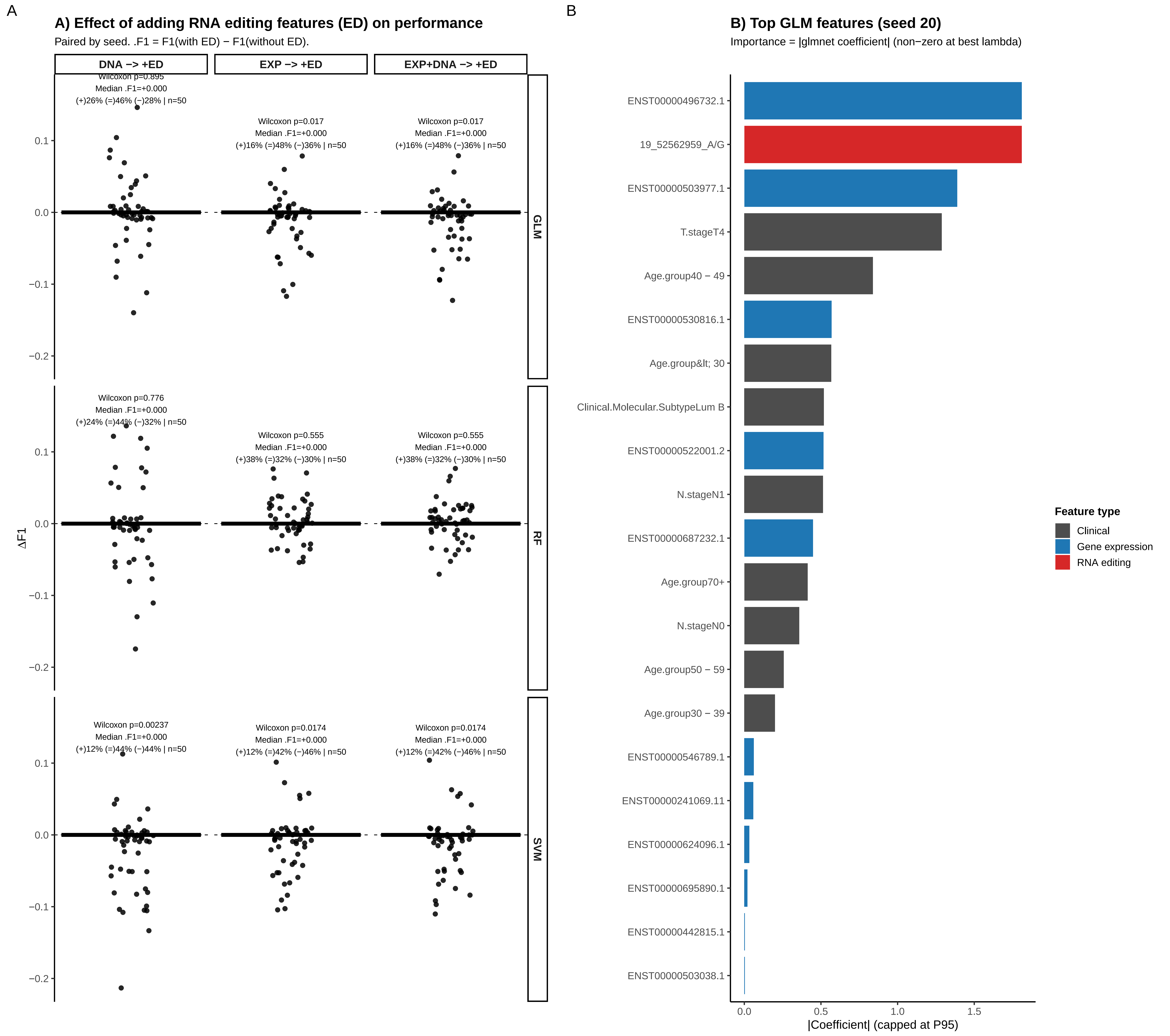

A
A) Effect of adding RNA editing features (ED) on performance
Paired by seed. ΔF1 = F1(with ED) − F1(without ED).
DNA −> +ED
EXP −> +ED
EXP+DNA −> +ED
GLM
RF
SVM
ΔF1
Wilcoxon p=0.895
Median ΔF1=+0.000
(+)26% (=)46% (−)28% | n=50
Wilcoxon p=0.017
Median ΔF1=+0.000
(+)16% (=)48% (−)36% | n=50
Wilcoxon p=0.017
Median ΔF1=+0.000
(+)16% (=)48% (−)36% | n=50
Wilcoxon p=0.776
Median ΔF1=+0.000
(+)24% (=)44% (−)32% | n=50
Wilcoxon p=0.555
Median ΔF1=+0.000
(+)38% (=)32% (−)30% | n=50
Wilcoxon p=0.555
Median ΔF1=+0.000
(+)38% (=)32% (−)30% | n=50
Wilcoxon p=0.00237
Median ΔF1=+0.000
(+)12% (=)44% (−)44% | n=50
Wilcoxon p=0.0174
Median ΔF1=+0.000
(+)12% (=)42% (−)46% | n=50
Wilcoxon p=0.0174
Median ΔF1=+0.000
(+)12% (=)42% (−)46% | n=50
0.1
0.0
−0.1
−0.2
B
B) Top GLM features (seed 20)
Importance = |glmnet coefficient| (non−zero at best lambda)
ENST00000496732.1
19_52562959_A/G
ENST00000503977.1
T.stageT4
Age.group40 − 49
ENST00000530816.1
Age.group< 30
Clinical.Molecular.SubtypeLum B
ENST00000522001.2
N.stageN1
ENST00000687232.1
Age.group70+
N.stageN0
Age.group50 − 59
Age.group30 − 39
ENST00000546789.1
ENST00000241069.11
ENST00000624096.1
ENST00000695890.1
ENST00000442815.1
ENST00000503038.1
0.0
0.5
1.0
1.5
|Coefficient| (capped at P95)
Feature type
Clinical
Gene expression
RNA editing
