# Supplementary figures and images for: Integration of RNA Editing into Multiomics Machine Learning Models for Predicting Drug Responses in Breast Cancer Patients
Source: Biomedicines. 2026 Mar 14;14(3):665. doi: 10.3390/biomedicines14030665 (PMC13024426; doi:10.3390/biomedicines14030665)

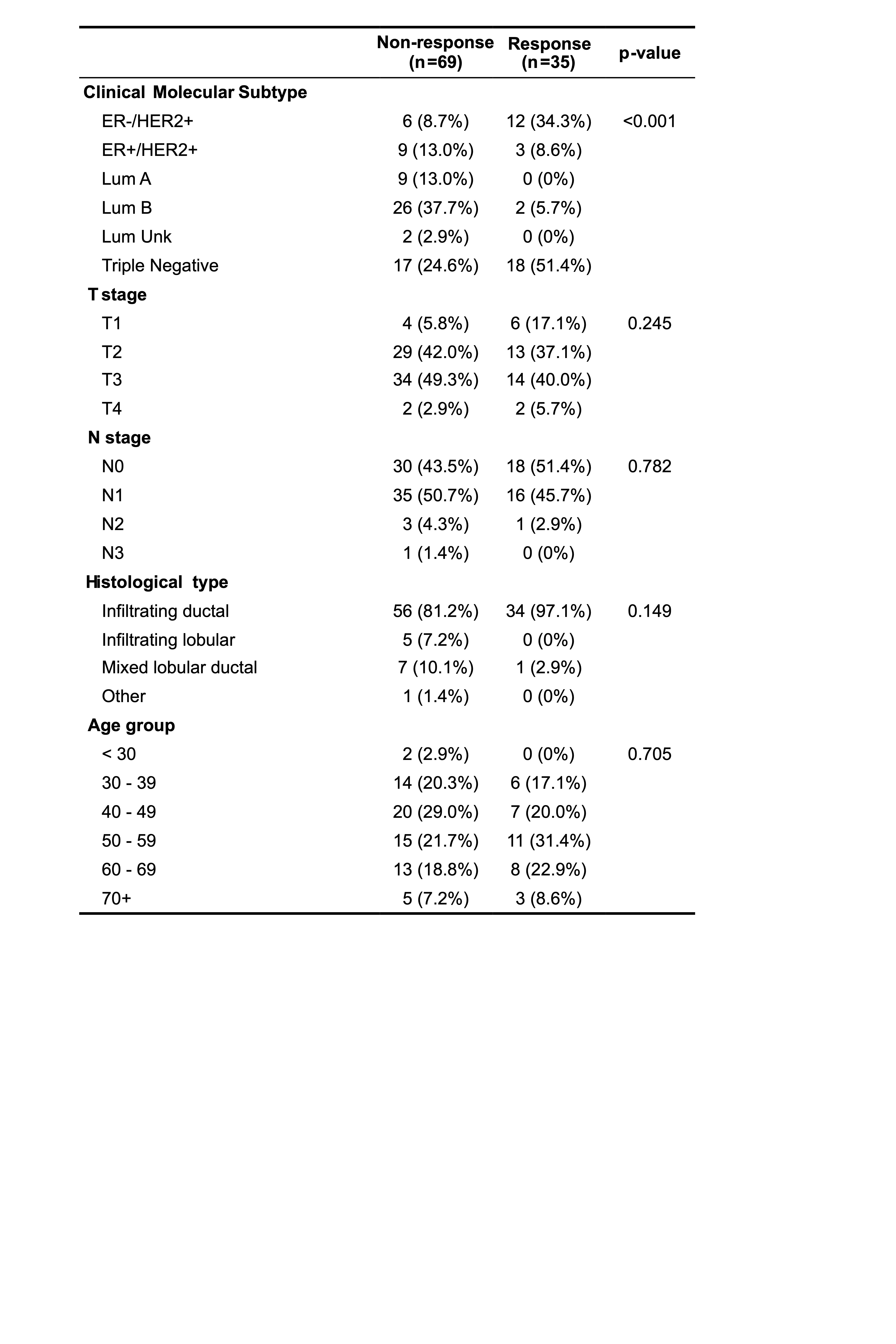

Supplement: Supplementary file 1 [file biomedicines-14-00665-s001.zip › Figure S1.jpg]

Model performance across datasets

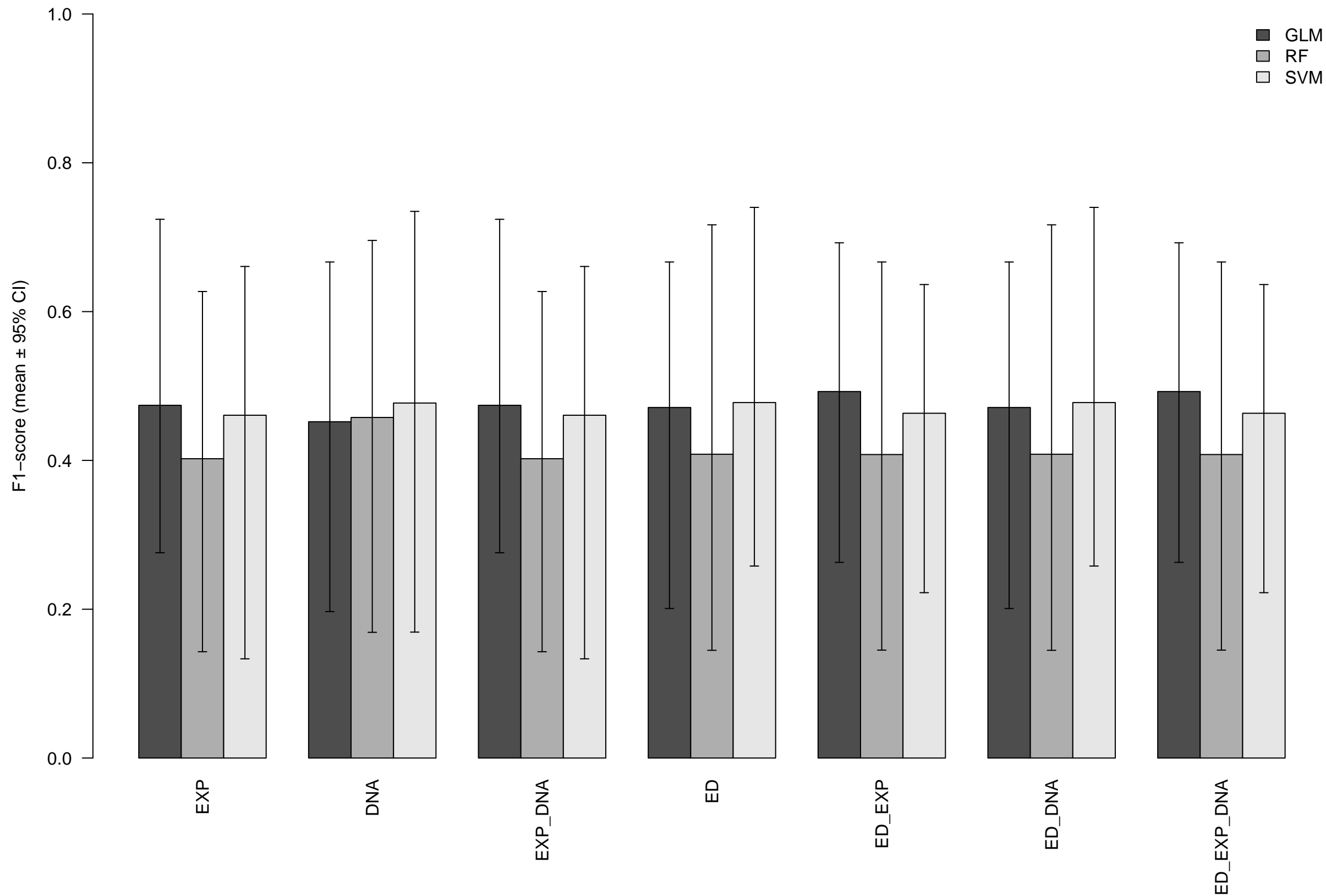

Supplement: Supplementary file 1 [file biomedicines-14-00665-s001.zip › Figure S2.pdf]

Model performance across 50 train/test splits

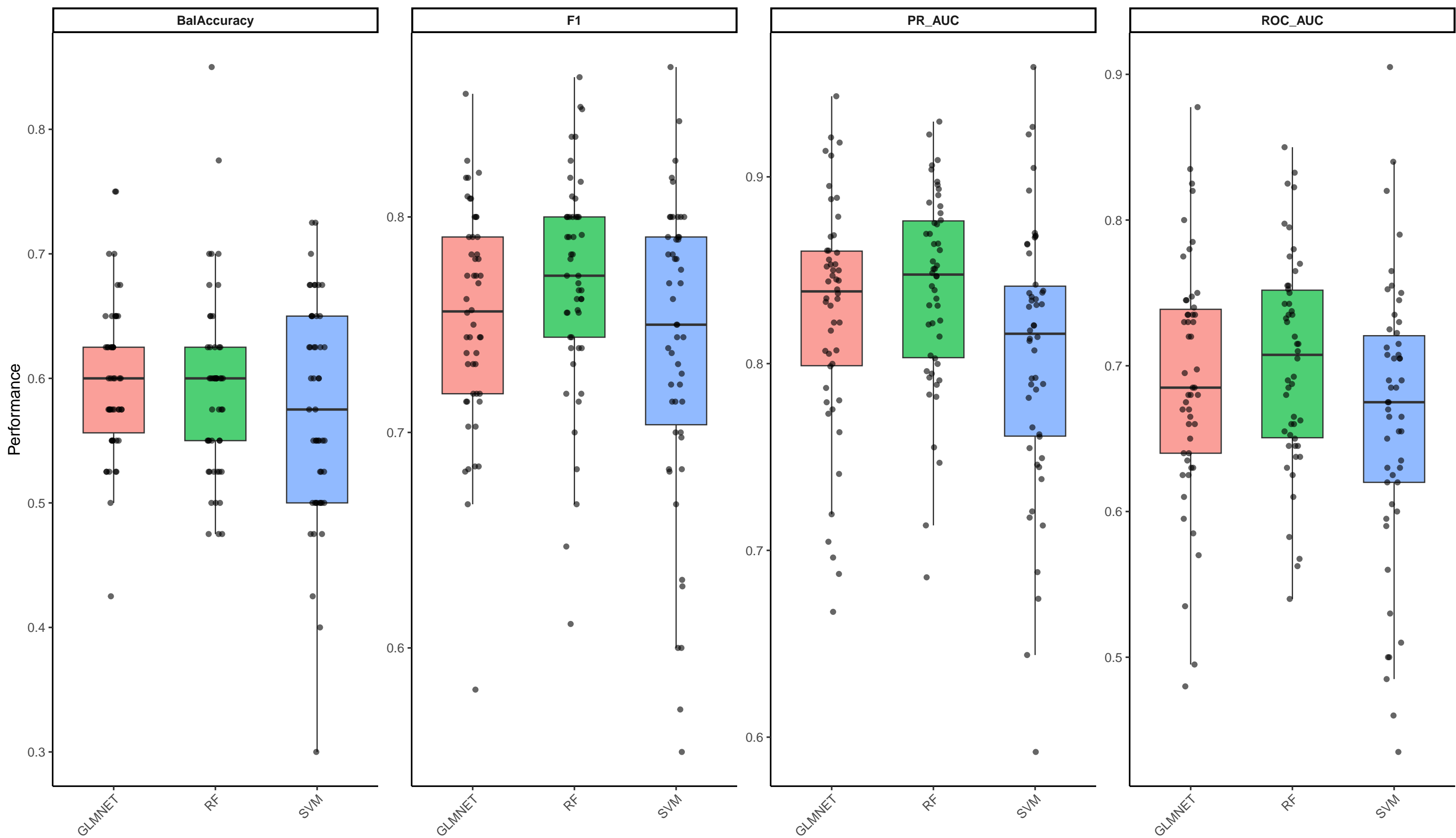

Supplement: Supplementary file 1 [file biomedicines-14-00665-s001.zip › Figure S4.pdf]

MCC

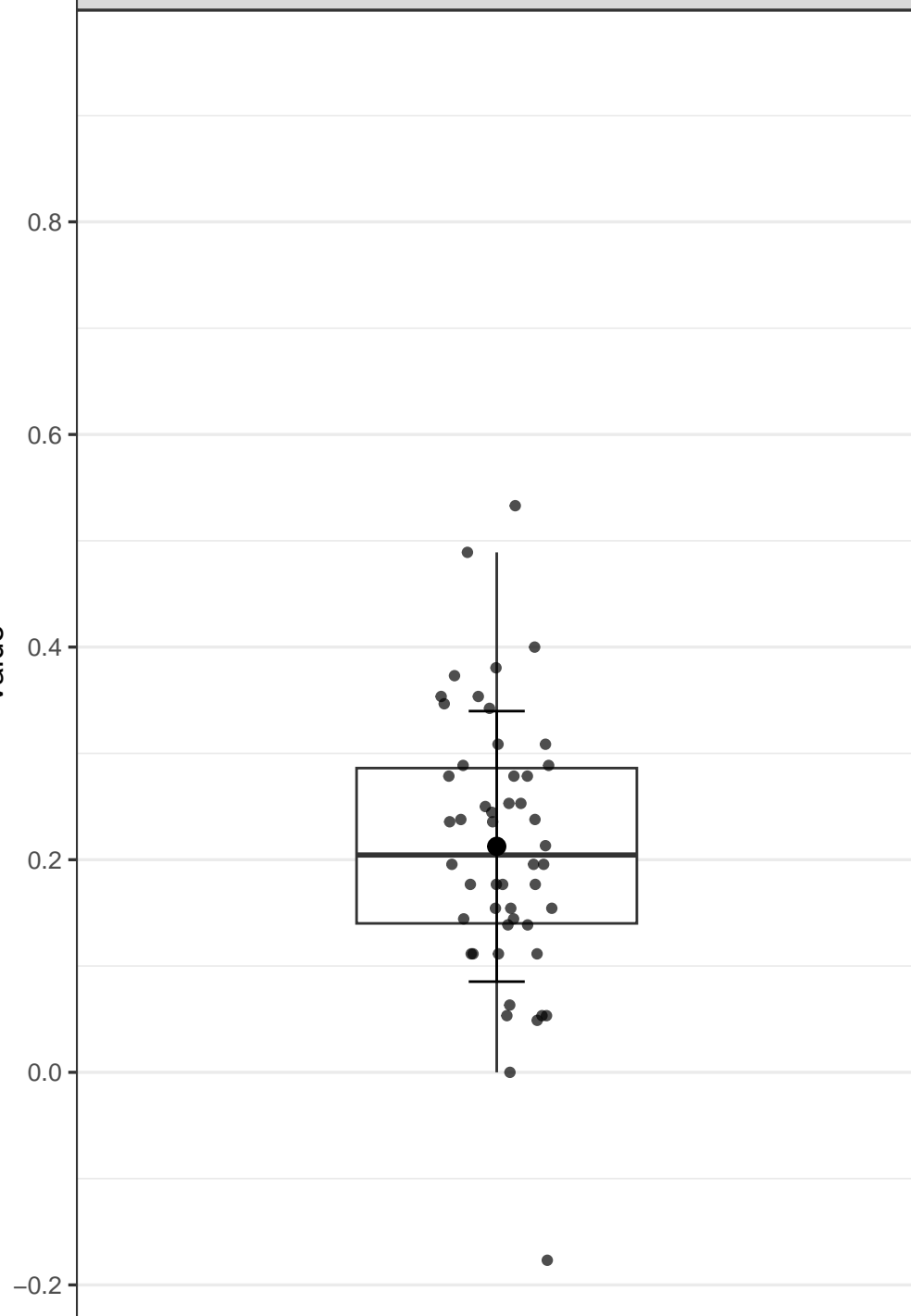

PR\_AUC

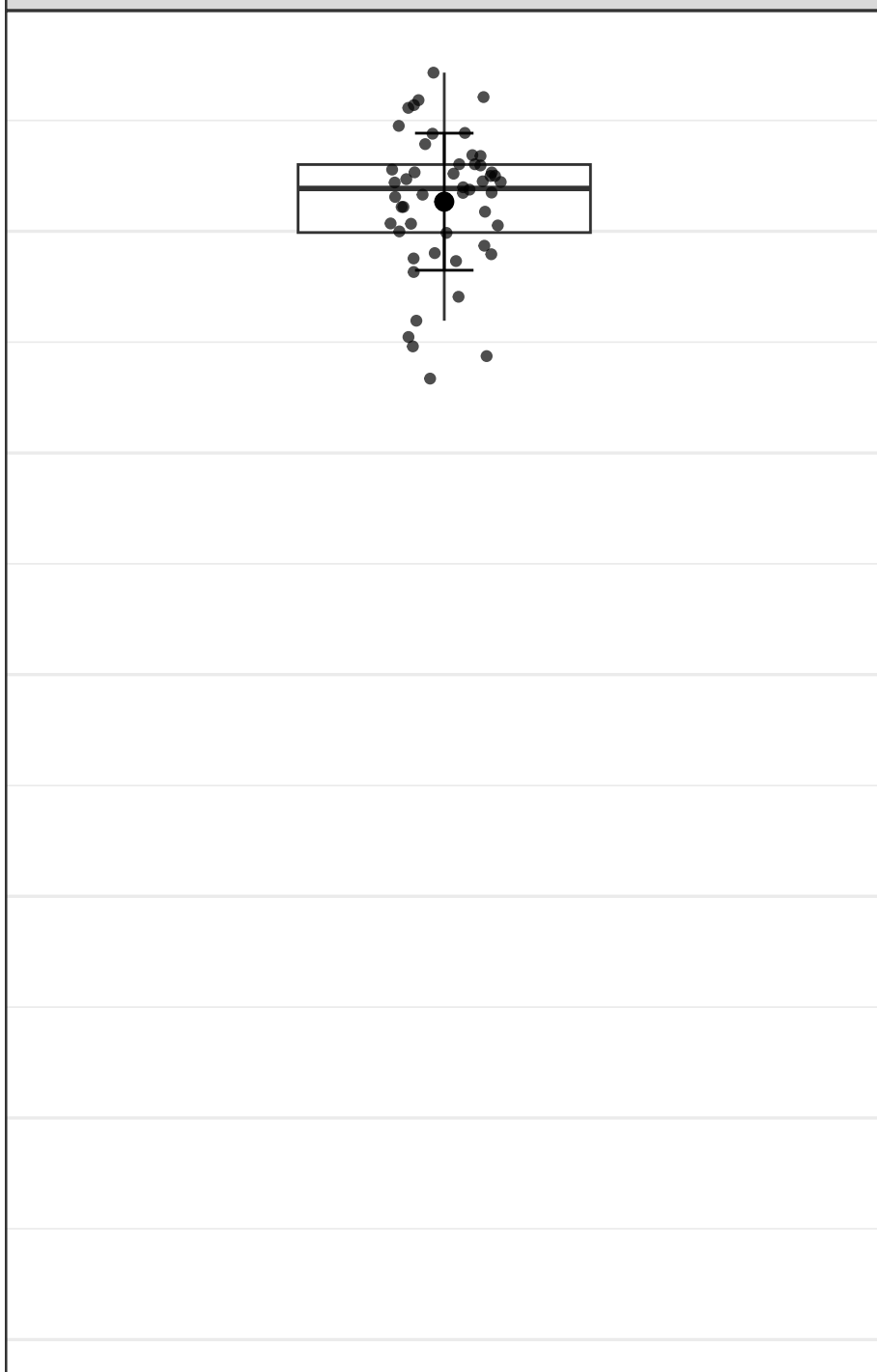

Supplement: Supplementary file 1 [file biomedicines-14-00665-s001.zip › Figure S5.pdf]
